# Simultaneous sensitive detection of multiple DNA glycosylases from lung cancer cells at the single-molecule level†
†Electronic supplementary information (ESI) available: Molecular mechanism of the DNA glycosylase-mediated cleavage of molecular beacons and optimization of the experimental conditions. See DOI: 10.1039/c7sc04296e


**DOI:** 10.1039/c7sc04296e

**Published:** 2017-11-07

**Authors:** Juan Hu, Ming-hao Liu, Ying Li, Bo Tang, Chun-yang Zhang

**Affiliations:** a College of Chemistry, Chemical Engineering and Materials Science , Collaborative Innovation Center of Functionalized Probes for Chemical Imaging in Universities of Shandong , Key Laboratory of Molecular and Nano Probes , Ministry of Education , Shandong Provincial Key Laboratory of Clean Production of Fine Chemicals , Shandong Normal University , Jinan 250014 , China . Email: cyzhang@sdnu.edu.cn ; Email: tangb@sdnu.edu.cn ; Fax: +86 531 82615258 ; Fax: +86 531 86180017 ; Tel: +86 531 86186033 ; Tel: +86 531 86180010; b School of Medicine , Health Science Center , Shenzhen University , Shenzhen 518060 , China

## Abstract

DNA glycosylases are involved in the base excision repair pathway, and all mammals express multiple DNA glycosylases to maintain genome stability. However, the simultaneous detection of multiple DNA glycosylase still remains a great challenge. Here, we develop a single-molecule detection method for the simultaneous detection of human 8-oxoguanine DNA glycosylase 1 (hOGG1) and human alkyladenine DNA glycosylase (hAAG) on the basis of DNA glycosylase-mediated cleavage of molecular beacons. We designed a Cy3-labeled molecular beacon modified with 8-oxoguanine (8-oxoG) for a hOGG1 assay and a Cy5-labeled molecular beacon modified with deoxyinosine for a hAAG assay. hOGG1 may catalyze the removal of 8-oxoG from 8-oxoG/C base pairs to generate an apurinic/apyrimidinic (AP) site, and hAAG may catalyze the removal of deoxyinosine from deoxyinosine/T base pairs to generate an AP site. With the assistance of apurinic/apyrimidinic endonuclease (APE1), the cleavage of AP sites results in the cleavage of molecular beacons, with Cy3 indicating the presence of hOGG1 and Cy5 indicating the presence of hAAG. Both of the Cy3 and Cy5 signals can be simply quantified by total internal reflection fluorescence-based single-molecule detection. This method can simultaneously detect multiple DNA glycosylases with a detection limit of 2.23 × 10^–6^ U μL^–1^ for hOGG1 and 8.69 × 10^–7^ U μL^–1^ for hAAG without the involvement of any target amplification. Moreover, this method can be used for the screening of enzyme inhibitors and the simultaneous detection of hOGG1 and hAAG from lung cancer cells, having great potential for further application in early clinical diagnosis.

## Abstract 2

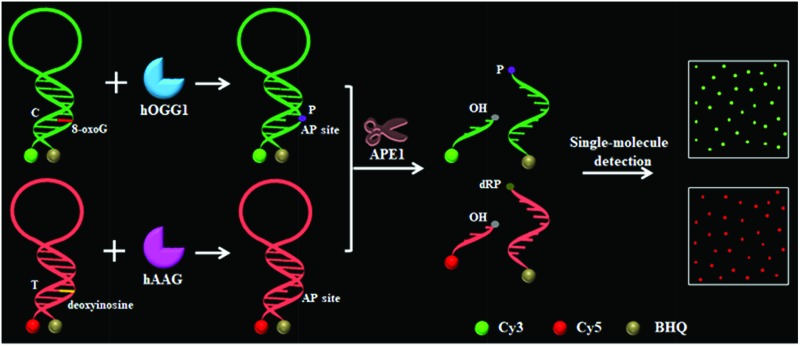
We demonstrate the simultaneous detection of human 8-oxoguanine DNA glycosylase 1 and human alkyladenine DNA glycosylase at the single-molecule level.

## Introduction

Base excision repair may correct DNA damage from alkylation, deamination and oxidation,^1^,^2^ and its repair pathway is initiated by one of at least 11 distinct mammalian DNA glycosylases in a lesion type-dependence manner.^3^ Moreover, aberrant DNA glycosylases are associated with a variety of diseases, such as cancers,^4^–^6^ neurological disease,^7^ cardiovascular disease^8^ and inflammation,^9^ suggesting the high potential of DNA glycosylases in cancer diagnosis and treatment.^10^,^11^ Lung cancer, with the highest mortality rate, is caused primarily by tobacco smoke. Recent research reveals that human 8-oxoguanine DNA glycosylase (hOGG1)^12^,^13^ and human alkyladenine DNA glycosylase (hAAG)^14^ may become biomarkers for lung cancer risk assessment and prevention. The bi-functional hOGG1 enzyme is responsible for the excision of 8-oxoguanine (8-oxoG) with combined glycosylase/lyase activity.^15^–^18^ hOGG1 excises 8-oxoG from the 8-oxoG/C base pairs so that other enzymes in the BER pathway can subsequently restore the G/C base pairs. The mono-functional hAAG enzyme exhibits broad substrate specificity and is responsible for the recognition and excision of a diverse group of alkylated purine bases (*e.g.* 3-methyladenine, 7-methylguanine and 1-*N*^6^-ethenoadenine) and the removal of hypoxanthine from deoxyinosine-containing DNA (Fig. S1, ESI†).^19^,^20^ Therefore, the simultaneous measurement of hOGG1 and hAAG activities is of great importance for the clinical diagnosis of lung cancer.

Conventional methods for DNA glycosylase (*e.g.* hOGG1 and hAAG) assay include radioactive labeling, enzyme-linked immunosorbent assay, high-performance liquid chromatography,^13^ magnetic nanoparticle-based separation techniques,^21^ gold nanoparticle-based colorimetric assay,^22^,^23^ and electrochemiluminescent^24^ and fluorescent methods.^25^ However, these methods suffer from some limitations, such as the involvement of costly labeling reagents, low specificity, tedious DNA fragmentation and expensive instrumentation,^13^ long analysis time and complicated procedures,^21^–^23^ and low detection sensitivity.^22^–^25^ To overcome these limitations, several amplification strategies have been introduced, including exonuclease (*e.g.* lambda exonuclease and exonuclease III)-assisted signal amplification,^26^,^27^ target-induced autocatalytic DNAzyme-generated rolling circle amplification,^28^ and the use of a lower denaturation temperature polymerase chain reaction.^29^ However, they usually involve some special requirements, such as the use of a special exonuclease,^26^,^27^ the ligation of a padlock probe,^28^ high-precision thermal cycling, and the use of multiple primers and special DNA polymerases,^29^ inevitably increasing the experimental complexity and cost. In addition, the reported amplification methods enable the detection of only a single type of DNA glycosylase.^27^–^29^ Therefore, the development of a simple and sensitive method for the simultaneous detection of multiple DNA glycosylases still remains a great challenge.

In this research, we develop a sensitive single-molecule detection method for the simultaneous detection of hOGG1 and hAAG from lung cancer cells on the basis of the DNA glycosylase-mediated cleavage of molecular beacons. In comparison with the ensemble measurement, single-molecule detection has distinct advantages of ultrahigh sensitivity, rapidity, simplicity, high signal-to-noise ratio and low sample consumption,^30^ and has been applied for the sensitive detection of DNA,^31^ microRNA,^32^ proteins^33^,^34^ and cancer cells^35^ at the single-molecule level. We designed a Cy3-labeled molecular beacon modified with 8-oxoG for a hOGG1 assay and a Cy5-labeled molecular beacon modified with deoxyinosine for a hAAG assay. In contrast to the conventional molecular beacons which are strongly affected by thermodynamics and kinetics,^36^ the restoration of Cy3 and Cy5 fluorescence is induced by the DNA glycosylase-mediated cleavage of molecular beacons, with Cy3 indicating the presence of hOGG1 and Cy5 indicating the presence of hAAG. Both of the Cy3 and Cy5 signals can be simply quantified by total internal reflection fluorescence (TIRF)-based single-molecule detection. This method can simultaneously detect multiple DNA glycosylases with a detection limit of 2.23 × 10^–6^ U μL^–1^ for hOGG1 and 8.69 × 10^–7^ U μL^–1^ for hAAG without the involvement of any target amplification, and it can be used for the simultaneous measurement of enzyme kinetic parameters and the detection of hOGG1 and hAAG activities from lung cancer cells.

## Results and discussion

### Principles of the multiple DNA glycosylase assay

**Scheme 1:**
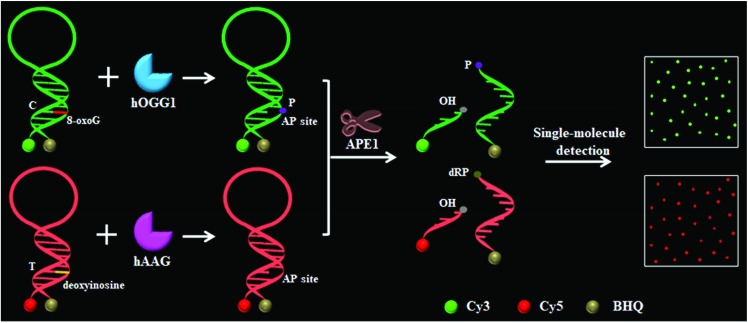
Schematic illustration of the multiple DNA glycosylase assay using the DNA glycosylase-mediated cleavage of molecular beacons and single-molecule detection.

To demonstrate the simultaneous detection of multiple DNA glycosylases, we used hOGG1 and hAAG as model enzymes. hOGG1 and hAAG may initiate the first step of base excision repair, and are considered to be functional biomarkers for lung cancer.^12^–^14^ The principle of the DNA glycosylase assay is illustrated in Scheme 1. This assay involves two steps: (1) the DNA glycosylase-mediated cleavage of molecular beacons and (2) the subsequent single-molecule detection. We designed two specific substrates for hOGG1 and hAAG, respectively. The substrate for hOGG1 is labeled with a Cy3 fluorophore at the 5′ terminus and a BHQ2 quencher at the 3′ terminus, and is modified with 8-oxoG positioned 6 deoxynucleotides downstream of a 5′-terminus. The substrate for hAAG is labeled with a Cy5 fluorophore at the 5′ terminus and a BHQ3 quencher at the 3′ terminus, and is modified with deoxyinosine positioned 5 deoxynucleotides downstream of a 5′-terminus (Scheme 1). The substrate for hOGG1 and the substrate for hAAG were used to prepare the molecular beacons, respectively, with the Cy3 fluorescence being quenched by BHQ2 and the Cy5 fluorescence being quenched by BHQ3 as a result of the fluorescence resonance energy transfer (FRET) effect.^36^ The Cy3-labeled molecular beacon may be recognized by hOGG1 because 8-oxoG forms a base pair with cytosine upon the formation of a double-stranded DNA stem, and the Cy5-labeled molecular beacon may be recognized by hAAG because deoxyinosine forms a base pair with thymine upon the formation of a double-stranded DNA stem (Fig. S1, ESI†). In contrast to conventional molecular beacons with stem sequences of 5–7 base pairs in length, the stem lengths of the Cy3-labeled molecular beacon and the Cy5-labeled molecular beacon may be increased to 12–13 base pairs. Since the restoration of Cy3 and Cy5 fluorescence is induced by the DNA glycosylase-mediated cleavage of molecular beacons, the longer stem length can improve the selectivity of the molecular beacons for a DNA glycosylase assay. In addition, the longer stem length may lead to a lower fluorescence background, facilitating the improvement of detection sensitivity.^36^ In the presence of hOGG1, dual molecular beacons and the APE1 enzyme, hOGG1 recognizes the 8-oxoG/C base pairs and cleaves the *N*-glycosidic bond between the sugar and the damaged base, releasing the damaged base to form an AP site.^15^–^17^ The AP site is then cleaved by a Schiff base intermediate and APE1, leading to the cleavage of the Cy3-labeled molecular beacon into two portions (*i.e.* a Cy3-labeled DNA fragment with a hydroxyl (OH) terminus and a BHQ2-labeled DNA fragment with a phosphate (P) terminus) (Fig. S2A, ESI†). The spatial separation of the fluorophore from the quencher results in the restoration of Cy3 fluorescence which can be sensitively quantified by single-molecule detection, with Cy3 indicating the presence of hOGG1. Similarly, in the presence of hAAG, dual molecular beacons and the APE1 enzyme, hAAG recognizes deoxyinosine/T base pairs and cleaves the *N*-glycosidic bond between the sugar and the damaged base, releasing the damaged base to form an AP site.^19^,^20^ APE1 then cleaves the AP site, leading to the cleavage of the Cy5-labeled molecular beacon into two portions (*i.e.* a Cy5-labeled DNA fragment with an OH terminus and a BHQ3-labeled DNA fragment with a deoxyribose-phosphate (dRP) terminus) (Fig. S2B, ESI†). The spatial separation of the fluorophore from the quencher results in the restoration of Cy5 fluorescence, which can be sensitively quantified by single-molecule detection, with Cy5 indicating the presence of hAAG. When both hOGG1 and hAAG are present, 8-oxoG and deoxyinosine may be removed from the dual molecular beacons by hOGG1 and hAAG, respectively, leading to the formation of AP sites whose cleavage results in the restoration of both Cy3 and Cy5 fluorescence. However, in the absence of hOGG1 and hAAG, neither the 8-oxoG base nor deoxyinosine can be removed, and no AP site is formed. As a result, no AP site cleavage occurs, and both Cy3 and Cy5 are still caged in the molecular beacons and neither Cy3 nor Cy5 fluorescence is observed.

### Validation of the assay

**Fig. 1:**
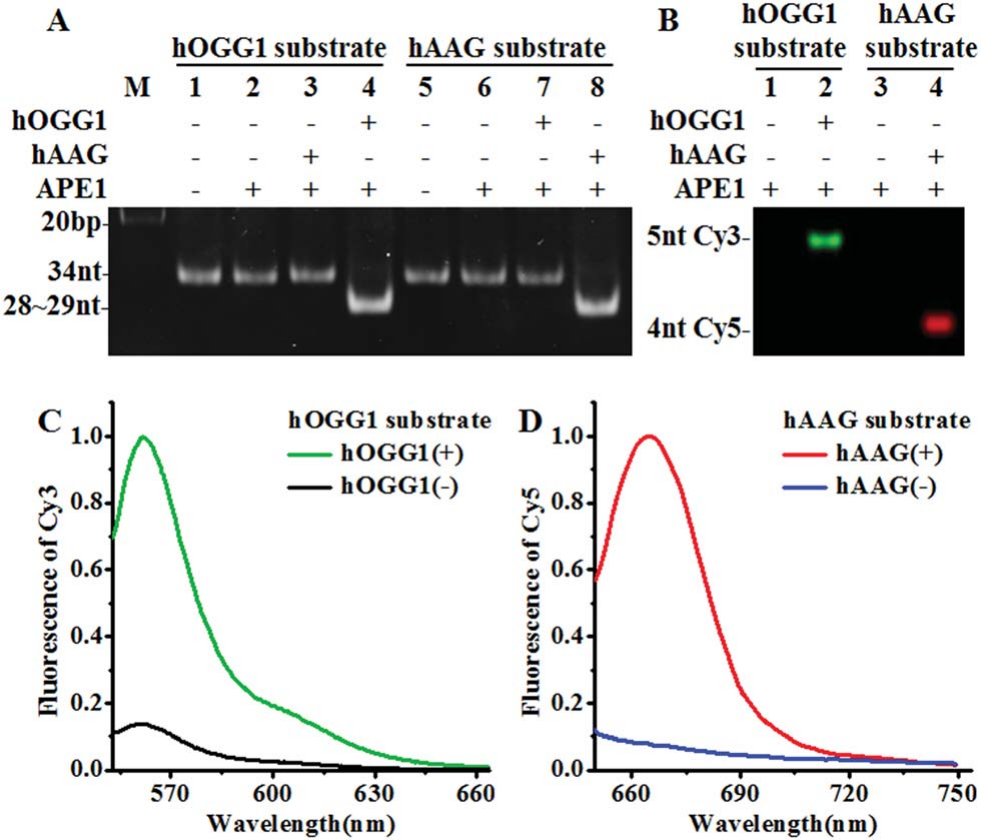
(A) PAGE analysis of the hOGG1-mediated cleavage of the Cy3-labeled molecular beacon (lanes 1–4) and the hAAG-mediated cleavage of the Cy5-labeled molecular beacon (lanes 5–8) with SYBR Gold as the indicator. (B) PAGE analysis of the hOGG1-mediated cleavage of the Cy3-labeled molecular beacon and the hAAG-mediated cleavage of the Cy5-labeled molecular beacon by excitation of Cy3 and Cy5. The green color indicates the Cy3-labeled DNA fragment in the presence of hOGG1 (lane 2) and the red color indicates the Cy5-labeled DNA fragment in the presence of hAAG (lane 4). (C) Fluorescence measurements of the hOGG1-mediated cleavage of the Cy3-labeled molecular beacon in the absence (black line) and presence (green line) of hOGG1. (D) Fluorescence measurements of the hAAG-mediated cleavage of the Cy5-labeled molecular beacon in the absence (blue line) and presence (red line) of hAAG. The hOGG1 concentration is 0.1 U μL^–1^ and the hAAG concentration is 0.1 U μL^–1^, and the APE1 concentration is 0.1 U μL^–1^.

We designed a Cy3-labeled molecular beacon modified with 8-oxoG for a hOGG1 assay and a Cy5-labeled molecular beacon modified with a deoxyinosine for a hAAG assay. We used non-denaturating polyacrylamide gel electrophoresis (PAGE) and fluorescence measurements to verify the assay feasibility. As shown in Fig. 1A, the enzyme reaction products were analyzed by PAGE with SYBR Gold as the indicator. In the absence of DNA glycosylases (Fig. 1A, lanes 1, 2, 5 and 6), only the 34 nt band from the Cy3-labeled molecular beacon/Cy5-labeled molecular beacon was observed, indicating no occurrence of the cleavage reaction. In the presence of the Cy3-labeled molecular beacon + hOGG1 + APE1, a distinct band of 28 nt was observed (Fig. 1A, lane 4 and Fig. S2C†), indicating the hOGG1-mediated base removal and the cleavage of the Cy3-labeled molecular beacon. Similarly, in the presence of the Cy5-labeled molecular beacon + hAAG + APE1, a distinct band of ∼29 nt was observed (Fig. 1A, lane 8 and Fig. S2D†), indicating the hAAG-mediated base removal and the cleavage of the Cy5-labeled molecular beacon. In contrast, no band of 28–29 nt was observed in the presence of either the Cy3-labeled molecular beacon + hAAG + APE1 (Fig. 1A, lane 3) or the Cy5-labeled molecular beacon + hOGG1 + APE1 (Fig. 1A, lane 7). These results were further confirmed by PAGE analysis with the direct excitation of Cy3 and Cy5 (Fig. 1B). No band was observed in the presence of either the Cy3-labeled molecular beacon + APE1 (Fig. 1B, lane 1) or the Cy5-labeled molecular beacon + APE1 (Fig. 1B, lane 3) due to no occurrence of the cleavage reaction and the quenching of the fluorophores by quenchers. In the presence of hOGG1, a distinct band of 5 nt resulting from the Cy3-labeled DNA fragment was observed (Fig. 1B, lane 2 and Fig. S2C†), indicating the hOGG1-mediated base removal and the cleavage of the Cy3-labeled molecular beacon. In the presence of hAAG, a distinct band of 4 nt resulting from the Cy5-labeled DNA fragment was observed (Fig. 1B, lane 4 and Fig. S2D†), indicating the hAAG-mediated base removal and the cleavage of the Cy5-labeled molecular beacon. The results of PAGE analysis are consistent with those of the fluorescence measurements (Fig. 1C and D). In the control experiment without the DNA glycosylases, neither a significant Cy3 signal (Fig. 1C, black line) nor a significant Cy5 signal (Fig. 1D, blue line) was detected. In contrast, a distinct Cy3 fluorescence signal with a characteristic emission peak of 562 nm was observed in the presence of hOGG1 (Fig. 1C, green line), and a distinct Cy5 fluorescence signal with a characteristic emission peak of 665 nm was observed in the presence of hAAG (Fig. 1D, red line). These results clearly demonstrate that the proposed method can be used for the simultaneous detection of hOGG1 and hAAG.

**Fig. 2:**
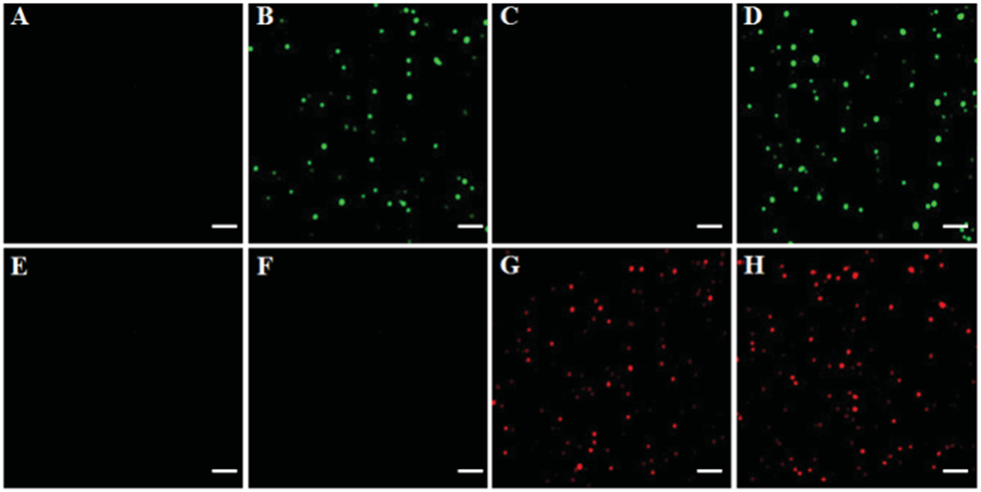
Simultaneous detection of multiple DNA glycosylases by TIRF-based single-molecule imaging in the absence (A and E) and presence of hOGG1 (B and F), hAAG (C and G) and both hOGG1 and hAAG (D and H). The Cy3 fluorescence signals are shown in green, and the Cy5 fluorescence signals are shown in red. The Cy3-labeled molecular beacon (0.3 μM), the Cy5-labeled molecular beacon (0.3 μM), hOGG1 (0.1 U μL^–1^), hAAG (0.1 U μL^–1^) and APE1 (0.1 U μL^–1^) were used in this research. The scale bar is 5 μm.

To investigate the feasibility of the proposed method for multiple DNA glycosylase assays, we simultaneously measured hOGG1 and hAAG at the single-molecule level. As shown in Fig. 2, there was neither a Cy3 (Fig. 2A) nor a Cy5 fluorescence signal (Fig. 2E) in the absence of hOGG1 and hAAG. In contrast, distinct Cy3 fluorescence signals were observed in the presence of hOGG1 (Fig. 2B, green color), but no Cy5 fluorescence signal was obtained (Fig. 2F). In the presence of hAAG, distinct Cy5 fluorescence signals were observed (Fig. 2G, red color), but no Cy3 fluorescence signal was obtained (Fig. 2C). When both hOGG1 and hAAG were present, distinct Cy3 (Fig. 2D, green color) and Cy5 fluorescence signals (Fig. 2H, red color) were simultaneously observed. These results clearly demonstrate that the proposed method can be used for the simultaneous detection of multiple DNA glycosylases.

### Detection sensitivity

**Fig. 3:**
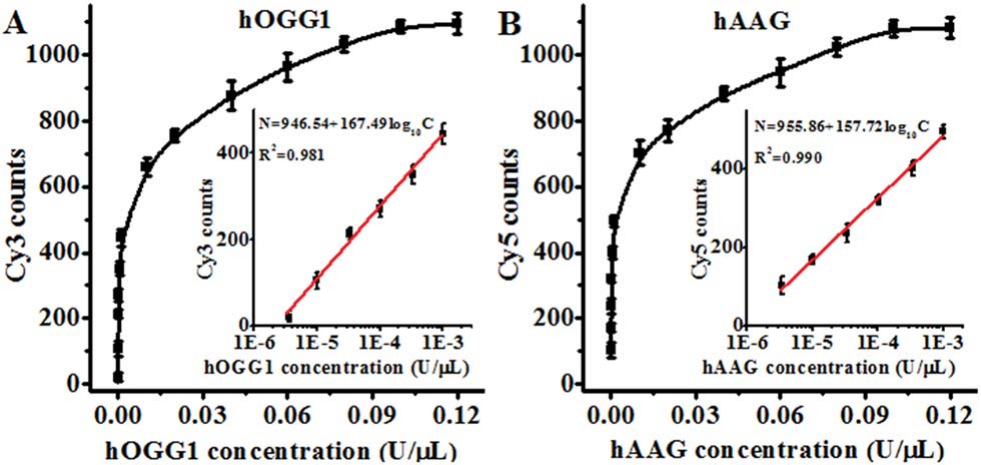
(A) Measurement of the Cy3 counts generated by different concentrations of hOGG1. The inset shows the linear relationship between the Cy3 counts and the logarithm of the hOGG1 concentration. The Cy3-labeled molecular beacon (0.3 μM) and APE1 (0.1 U μL^–1^) were used in this research. (B) Measurement of the Cy5 counts generated by different concentrations of hAAG. The inset shows the linear relationship between the Cy5 counts and the logarithm of the hAAG concentration. The Cy5-labeled molecular beacon (0.3 μM) and APE1 (0.1 U μL^–1^) were used in this research. The error bars represent the standard deviations of the three experiments.

Under the optimal experimental conditions (Fig. S3, ESI†), we investigated the sensitivity of the proposed method by measuring the variance of fluorescent counts with the concentration of DNA glycosylase. As shown in Fig. 3A, the Cy3 counts increase with an increasing concentration of hOGG1 from 3.4 × 10^–6^ to 0.1 U μL^–1^. In the logarithmic scale, the Cy3 counts show a linear correlation with the concentration of hOGG1 over a range of 3.4 × 10^–6^ to 1 × 10^–3^ U μL^–1^. The regression equation is *N* = 946.54 + 167.49 log_10_ *C* for hOGG1 (*R*^2^ = 0.981), where *N* is the measured Cy3 counts and *C* is the concentration of hOGG1, respectively. The detection limit is calculated to be 2.23 × 10^–6^ U μL^–1^ by evaluating the average response of the control group plus three times the standard deviation. The sensitivity of the proposed method was improved by as much as 2 orders of magnitude compared to that of the graphene/gold nanoparticle hybrid-based colorimetric assay (1.6 × 10^–3^ U μL^–1^)^23^ and that of the gold nanoparticle-based colorimetric assay (7 × 10^–4^ U μL^–1^),^22^ and it is comparable to that of the exonuclease-assisted isothermal amplification-based fluorescent assay (3.5 × 10^–6^ U μL^–1^ (ref. 26) and 1 × 10^–6^ U μL^–1^ (ref. 27)) and that of the rolling circle amplification-based fluorescent assay (1 × 10^–6^ U μL^–1^),^28^ even without the involvement of any target amplification. As shown in Fig. 3B, the Cy5 counts increase with an increasing concentration of hAAG from 3.4 × 10^–6^ to 0.1 U μL^–1^. In the logarithmic scale, the Cy5 counts have a linear correlation with the concentration of hAAG over a range of 3.4 × 10^–6^ to 1 × 10^–3^ U μL^–1^. The regression equation is *N* = 955.86 + 157.72 log_10_ *C* (*R*^2^ = 0.990), where *N* is the measured Cy5 counts and *C* is the concentration of hAAG, respectively. The detection limit is estimated to be 8.69 × 10^–7^ U μL^–1^ by evaluating the average response of the control group plus three times the standard deviation. The sensitivity of the proposed method was improved by as much as 2 orders of magnitude compared to that of the magnetic bead-based fluorescent assay (1 × 10^–4^ U μL^–1^).^21^ The improved sensitivity might be ascribed to (1) the specific hOGG1-induced 8-oxoG excision repair^15^–^17^ and hAAG-induced deoxyinosine excision repair,^19^,^20^ (2) the stimulation of hOGG1 activity by APE1 (ref. 37 and 38) and the activation of hAAG by APE1,^39^,^40^ (3) the low fluorescence background resulting from the designed molecular beacons with longer stem lengths than conventional molecular beacons,^36^ and (4) the high signal-to-noise ratio of single-molecule detection.^30^

### Detection selectivity

**Fig. 4:**
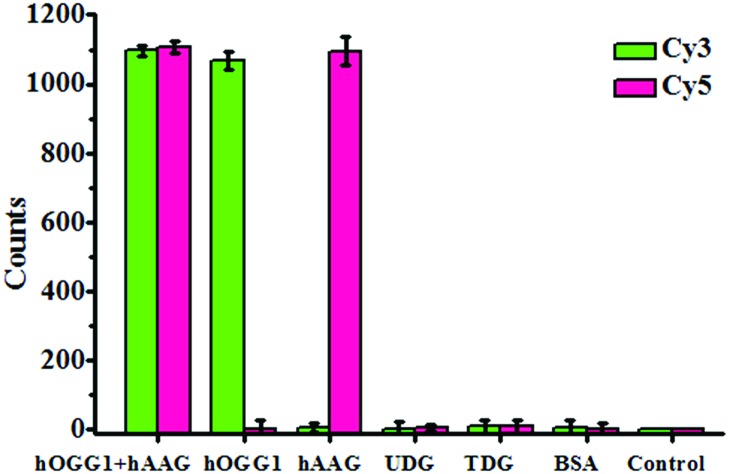
Measurement of the Cy3 counts and Cy5 counts in response to the reaction buffer (control), 0.1 g L^–1^ BSA, 0.1 U μL^–1^ UDG, 0.1 U μL^–1^ TDG, 0.1 U μL^–1^ hOGG1, 0.1 U μL^–1^ hAAG and 0.1 U μL^–1^ hOGG1 + 0.1 U μL^–1^ hAAG. The Cy3-labeled molecular beacon (0.3 μM), Cy5-labeled molecular beacon (0.3 μM) and APE1 (0.1 U μL^–1^) were used in this research. The error bars represent the standard deviations of the three experiments.

To investigate the selectivity of the proposed method for hOGG1 and hAAG assays, we used bovine serum albumin (BSA), uracil DNA glycosylase (UDG) and thymine DNA glycosylase (TDG) as negative controls. BSA is not a DNA glycosylase, and it cannot recognize the damaged bases. Thus, neither the Cy3 nor the Cy5 fluorescence signal can be observed in the presence of BSA (Fig. 4). Both UDG and TDG are mono-functional DNA glycosylases.^3^ Neither UDG nor TDG can recognize and cleave the Cy3-labeled molecular beacon and the Cy5-labeled molecular beacon. As a result, neither the Cy3 nor the Cy5 fluorescence signal can be observed in the presence of UDG and TDG, just like the control with only the reaction buffer (Fig. 4). In contrast, the addition of hOGG1 may induce a significant enhancement of the Cy3 fluorescence signal instead of the Cy5 fluorescence signal, while the addition of hAAG may induce a significant enhancement of the Cy5 fluorescence signal instead of the Cy3 fluorescence signal (Fig. 4). When both hOGG1 and hAAG co-exist, both of the Cy3 and Cy5 fluorescence signals can be simultaneously detected (Fig. 4). These results demonstrate the good selectivity of the proposed method towards hOGG1 and hAAG.

### Kinetic analysis

**Fig. 5:**
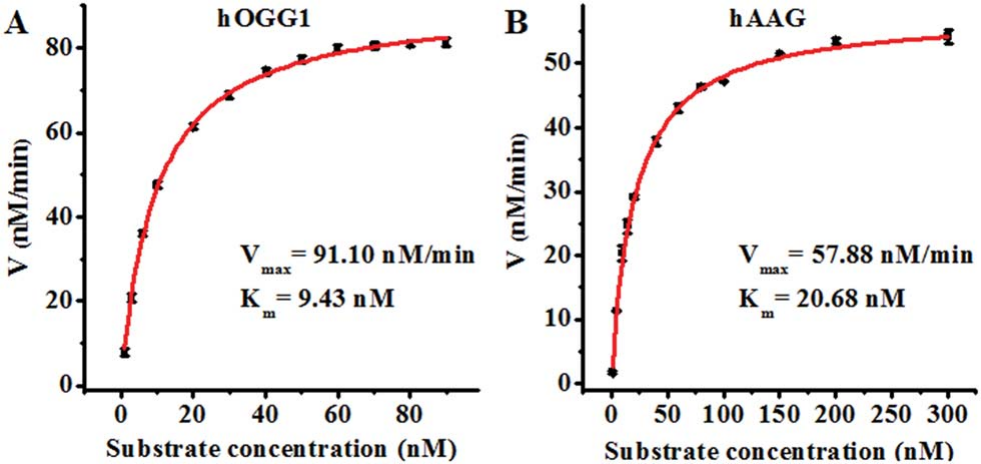
(A) Variance of the initial velocity in response to various concentrations of the Cy3-labeled molecular beacon substrate. The concentration of hOGG1 is 0.1 U μL^–1^ and the concentration of APE1 is 0.1 U μL^–1^. (B) Variance of the initial velocity in response to various concentrations of the Cy5-labeled molecular beacon substrate. The concentration of hAAG is 0.1 U μL^–1^ and the concentration of APE1 is 0.1 U μL^–1^. The error bars represent the standard deviations of the three experiments.

Since single-molecule detection may provide more accurate information on a single enzyme molecule, compared to typical fluorescence ensemble measurements that give only the average of the whole sample,^41^–^45^ we employed the proposed method to quantify the kinetic parameters at the single-molecule level. To evaluate the enzyme kinetic parameters of hOGG1, we measured the initial velocity in the presence of 0.1 U μL^–1^ hOGG1 and different concentrations of the Cy3-labeled molecular beacon for 2 min at 37 °C. To evaluate the enzyme kinetic parameters of hAAG, we measured the initial velocity in the presence of 0.1 U μL^–1^ hAAG and different concentrations of the Cy5-labeled molecular beacon for 5 min at 37 °C. As shown in Fig. 5, the initial velocities of hOGG1 (Fig. 5A) and hAAG (Fig. 5B) increase with the increasing concentration of the corresponding molecular beacons (*i.e.* DNA substrates). The experimental data are fitted to the Michaelis–Menten equation, *V* = *V*_max_[S]/(*K*_m_ + [S]), where *V*_max_ is the maximum initial velocity, [S] is the concentration of the molecular beacon and *K*_m_ is the Michaelis–Menten constant corresponding to the concentration at half-maximal velocity.^46^,^47^ *V*_max_ is calculated to be 91.10 nM min^–1^ and *K*_m_ is calculated to be 9.43 nM for hOGG1. The *K*_m_ value is consistent with that obtained by the radioactive assay (8.9 nM).^37^*V*_max_ is calculated to be 57.88 nM min^–1^ and *K*_m_ is calculated to be 20.68 nM for hAAG. The *K*_m_ value is consistent with that obtained by the radioactive assay (13–25 nM).^39^,^48^ These results suggest that the proposed method can be used to accurately evaluate the kinetic parameters of DNA glycosylases.

### Inhibition assay

**Fig. 6:**
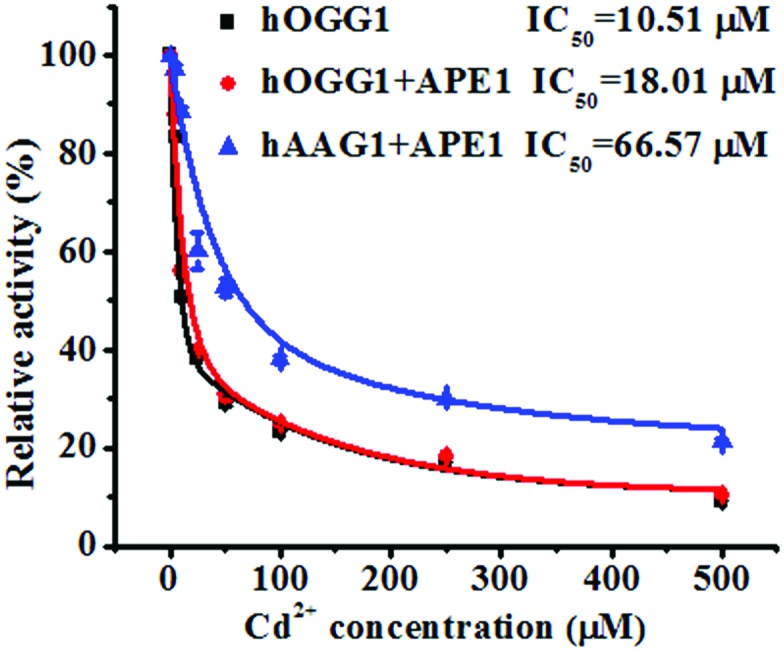
Variance of the relative catalytic activity with different concentrations of Cd^2+^ for hOGG1 alone (black line), hOGG1 + APE1 (red line) and hAAG + APE1 (blue line). The Cy3-labeled molecular beacon (0.3 μM), the Cy5-labeled molecular beacon (0.3 μM), hOGG1 (0.1 U μL^–1^), hAAG (0.1 U μL^–1^) and APE1 (0.1 U μL^–1^) were used in this research. The error bars represent the standard deviations of the three experiments.

To demonstrate the feasibility of the proposed method for an inhibition assay, we used cadmium (Cd^2+^) as a model inhibitor. Recent evidence suggests that Cd^2+^ might contribute to the increased risk of tumor formation in humans by interfering with and inhibiting the DNA repair processes.^49^,^50^ Cd^2+^ may induce the irreversible inactivation of hOGG1 by binding at Ca-binding sites in the structure of hOGG1 bound to its substrate,^15^,^51^,^52^ and Cd^2+^ exhibits the efficient inactivation of hAAG catalytic activity by occupying the Zn^2+^ binding sites of the hAAG active site.^53^ As shown in Fig. 6, the relative activities of hOGG1 and hAAG decrease with the increasing concentration of Cd^2+^. We used the IC_50_ value (half-maximal inhibitory concentration) to evaluate the inhibition effect of Cd^2+^ on DNA glycosylases. The IC_50_ value of hOGG1 alone is calculated to be 10.51 μM, consistent with the value of hOGG1 alone measured by the radioactive assay (∼10 μM).^51^,^52^ Interestingly, the IC_50_ value of hOGG1 in the presence of APE1 is evaluated to be 18.01 μM, much higher than the value of hOGG1 alone measured by the proposed method (10.51 μM) and by the radioactive assay (∼10 μM).^51^,^52^ Similarly, the IC_50_ value of hAAG in the presence of APE1 is evaluated to be 66.57 μM, much lower than the value of hAAG alone measured by radioactive assay (IC_50_ = ∼100 μM).^53^ The higher IC_50_ value of hOGG1 measured in the presence of APE1 and the lower IC_50_ value of hAAG measured in the presence of APE1 than those of the DNA glycosylases alone might be attributed to the inhibition of APE1 endonuclease activity by Cd^2+^ (note: the order of the measured IC_50_ values for different enzymes is as follows: hOGG1 (∼10 μM) < APE1 (26 μM) < hAAG (∼100 μM)).^54^ APE1 is an AP endonuclease and it initiates the repair of AP sites by incising the DNA backbone *via* a Mg^2+^-dependent reaction. Cd^2+^ may occupy two potential Mg^2+^ binding sites within the APE1 active site, resulting in the inactivation of APE1.^54^ These results clearly demonstrate that the proposed method may provide a new platform for the screening of DNA glycosylase inhibitors and the study of the DNA glycosylase inhibition mechanism.

### Real sample analysis

**Fig. 7:**
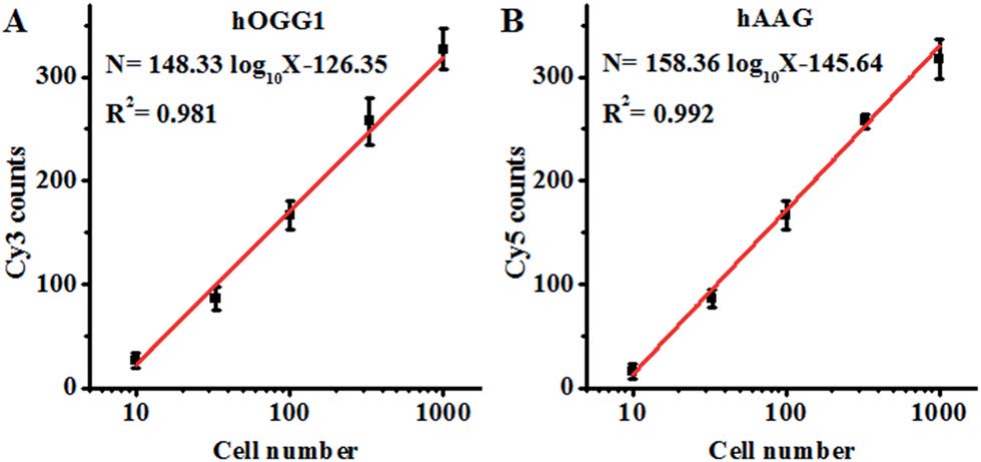
(A) Linear relationship between the Cy3 counts and the logarithm of the A549 cell number. (B) Linear relationship between the Cy5 counts and the logarithm of the A549 cell number. The Cy3-labeled molecular beacon (0.3 μM), the Cy5-labeled molecular beacon (0.3 μM) and APE1 (0.1 U μL^–1^) were used in this research. The error bars represent the standard deviations of the three experiments.

Both the hOGG1 and hAAG glycosylases are found in human lung cancer.^13^,^14^ To demonstrate the feasibility of the proposed method for a cellular DNA glycosylase assay, we simultaneously measured hOGG1 and hAAG from a lung adenocarcinoma cell line (A549 cells). As shown in Fig. 7A, the Cy3 counts increase with an increasing number of A549 cells, with a linear correlation being obtained between the Cy3 counts and the logarithm of the A549 cell number in the range of 10 to 1000 cells. The regression equation is *N* = 148.33 log_10_ *X* – 126.35 (*R*^2^ = 0.981), where *N* is the measured Cy3 counts and *X* is the number of A549 cells, respectively. The detection limit was calculated to be 7 cells by evaluating the average response of the control group plus three times the standard deviation, comparable to that obtained by the isothermal amplification-based fluorescent assay (4 cells)^26^ even without the involvement of any target amplification. These results suggest that the proposed method can be used to accurately quantify the cellular hOGG1 activity. As shown in Fig. 7B, the Cy5 counts increase with an increasing number of A549 cells, with a linear correlation being obtained between the Cy5 counts and the logarithm of the A549 cell number in the range of 10 to 1000 cells. The regression equation is *N* = 158.36 log_10_ *X* – 145.64 (*R*^2^ = 0.992), where *N* is the measured Cy5 counts and *X* is the number of A549 cells, respectively. The detection limit was calculated to be 9 cells by evaluating the average response of the control group plus three times the standard deviation. These results demonstrate that the proposed method can be used to accurately quantify the cellular hAAG activity.

## Conclusions

In summary, we have developed a single-molecule detection method for the simultaneous detection of hOGG1 and hAAG from lung cancer cells on the basis of the DNA glycosylase-mediated cleavage of molecular beacons. We designed a Cy3-labeled molecular beacon modified with 8-oxoG for a hOGG1 assay and a Cy5-labeled molecular beacon modified with deoxyinosine for a hAAG assay. In contrast to conventional molecular beacons which are strongly affected by thermodynamics and kinetics,^36^ the restoration of Cy3 and Cy5 fluorescence is induced by the DNA glycosylase-mediated cleavage of the molecular beacons, with Cy3 indicating the presence of hOGG1 and Cy5 indicating the presence of hAAG. Both of the Cy3 and Cy5 signals can be simply quantified by TIRF-based single-molecule detection. Owing to (1) the specific hOGG1-induced 8-oxoG excision repair^15^–^17^ and hAAG-induced deoxyinosine excision repair,^19^,^20^ (2) the stimulation of hOGG1 activity by APE1 (ref. 37 and 38) and the activation of hAAG by APE1,^39^,^40^ (3) the low fluorescence background resulting from the designed molecular beacons with longer stem lengths than conventional molecular beacons^36^ and (4) the high signal-to-noise ratio of single-molecule detection,^30^ the proposed method exhibits extremely high sensitivity with a detection limit of 2.23 × 10^–6^ U μL^–1^ for hOGG1 and 8.69 × 10^–7^ U μL^–1^ for hAAG, even without the involvement of any target amplification. The sensitivity of the proposed method was improved by as much as 2 orders of magnitude compared to that of the colorimetric assay^22^,^23^ and 2 orders of magnitude compared to that of the magnetic bead-based fluorescent assay,^21^ and is comparable to those of amplification-based approaches.^26^–^28^ This method can simultaneously detect multiple DNA glycosylases from lung cancer cells, and it can be used for the measurement of enzyme kinetic parameters and the screening of DNA glycosylase inhibitors, having great potential for further application in early clinical diagnosis and drug development. Importantly, this method can be extended to simultaneously detect other types of DNA repair enzymes by just using specific substrates for the preparation of molecular beacons.

## Experimental

### Materials

Human 8-oxoguanine DNA glycosylase (hOGG1), human alkyladenine DNA glycosylase (hAAG), human apurinic/apyrimidinic endonuclease 1 (APE1), 10× NEBuffer 2 (500 mM NaCl, 100 mM Tris–HCl, 100 mM MgCl_2_, 10 mM DTT, pH 7.9), 10× ThermoPol reaction buffer (200 mM Tris–HCl, 100 mM (NH_4_)_2_SO_4_, 100 mM KCl, 20 mM MgSO_4_, 1% Triton X-100, pH 8.8) and 10× NEBuffer 4 (500 mM potassium acetate, 200 mM Tris–acetate, 100 mM magnesium acetate, 10 mM DTT, pH 7.9) were purchased from New England Biolabs Inc. (Beverly, MA, U.S.A.). All HPLC-purified oligonucleotides (Table 1) were synthesized by Sangon Biotech Co., Ltd. (Shanghai, China).

**Table 1:** Sequences of the oligonucleotides^*a*^

Note	Sequence (5′–3′)
hOGG1 substrate	Cy3 – GGT CTO ATG GGG GAC ACG ACA CCC CCA TCA GAC C – BHQ2
hAAG substrate	Cy5 – CTC GIG GCA GCT CAG TAC AGG AAG CTG CCT CGA G – BHQ3

^*a*^The underlined letter “O” is 8-oxoG, and the underlined letter “I” is deoxyinosine.

### Formation of the molecular beacons and the enzyme reactions

Firstly, 6 μM hOGG1 substrate, 6 μM hAAG substrate and the mixture of 6 μM hOGG1 substrate and 6 μM hAAG substrate were incubated in 1× NEBuffer 2 (50 mM NaCl, 10 mM Tris–HCl, 10 mM MgCl_2_, 1 mM DTT, pH 7.9) at 95 °C for 5 min, respectively. After slowly cooling to room temperature, the products were placed on ice for further use. Then, 1 μL of the products of the molecular beacon (the final concentration of each substrate is 0.3 μM) were added into 20 μL of the reaction solution containing a varied concentration of hOGG1 and/or hAAG, 1× NEBuffer 2, 100 μg mL^–1^ BSA, 1× NEBuffer 4, 1× ThermoPol buffer and 0.1 U μL^–1^ APE1, followed by incubation at 37 °C for 1.5 h. The reaction was terminated by heating at 80 °C for 20 min.

### Gel electrophoresis and fluorescence measurements

The enzyme reaction products were analyzed by a Bio-Rad ChemiDoc MP Imaging System. The products stained with SYBR Gold were analyzed by 12% polyacrylamide gel electrophoresis (PAGE) in 1× TBE buffer (9 mM Tris–HCl, 9 mM boric acid, 0.2 mM EDTA, pH 7.9) at a 110 V constant voltage for 45 min at room temperature. The fluorescent DNA fragments of the enzyme reaction products were analyzed using an illumination source of Epi-green (520–545 nm excitation) and a 577–613 nm filter for the Cy3 fluorophores, and an illumination source of Epi-red (625–650 nm excitation) and a 675–725 nm filter for the Cy5 fluorophores. The fluorescence spectra of Cy3 and Cy5 were recorded by a Hitachi F-7000 fluorometer at the excitation wavelengths of 535 and 635 nm. The fluorescence intensity at the emission wavelengths of 562 and 665 nm was used for analysis.

### Single-molecule detection and data analysis

In the single-molecule measurement, the enzyme reaction products were further diluted 3000-fold with the buffer (10 mM Tris–HCl, 50 mM KCl, 5 mM MgCl_2_, 1 mM Trolox, pH 8.0). The 10 μL samples were spread on a glass coverslip for imaging. The images of the single molecules were acquired by TIRF microscopy (Nikon, Ti-E, Japan). Lasers of 561 nm and 640 nm were used to excite the Cy3 and Cy5 fluorescence simultaneously. The photons were collected using an oil immersion objective (CFI Apochromat TIRF 100×). The fluorescence was split up into the Cy3 channel (573–613 nm filter) and the Cy5 channel (661.5–690.5 nm filter) by a dichroic mirror, and was imaged onto a EMCCD camera (Photometrics, Evolve 512). For data analysis, a region of interest of 600 × 600 pixels was selected for Cy3 and Cy5 molecule counting using Image J software. The average Cy3 counts and average Cy5 counts were obtained by calculating ten frames.

### Inhibition assay

Varied concentrations of CdCl_2_ were incubated with 0.1 U μL^–1^ hOGG1 and 0.1 U μL^–1^ hAAG at 37 °C for 20 min, followed by incubation with the enzyme reaction mixture at 37 °C for 1.5 h. The reaction was terminated by heating at 80 °C for 20 min. The relative activity of the enzyme (RA) was measured according to
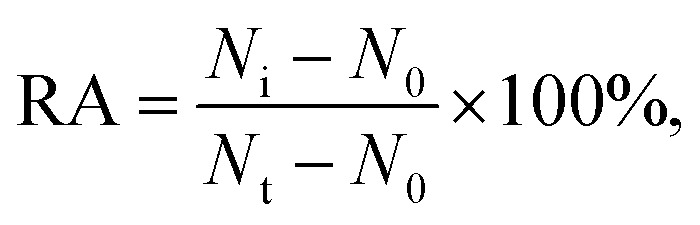
where *N*_0_ is the Cy3 counting number in the absence of hOGG1 or the Cy5 counting number in the absence of hAAG, *N*_t_ is the Cy3 counting number in the presence of hOGG1 (0.1 U μL^–1^) or the Cy5 counting number in the presence of hAAG (0.1 U μL^–1^), and *N*_i_ is the Cy3 counting number in the presence of both hOGG1 and Cd^2+^ or the Cy5 counting number in the presence of both hAAG and Cd^2+^. The IC_50_ value was calculated from the curve of RA *versus* the CdCl_2_ concentration.

### Cell culture and preparation of the cell extracts

The lung adenocarcinoma cell line (A549) was cultured in a DMEM medium (Invitrogen, U.S.A.) supplemented with 10% fetal bovine serum (Invitrogen, U.S.A.) and 1% penicillin–streptomycin at 37 °C under a 5% CO_2_ atmosphere. The nuclear extracts were prepared using a nuclear extract kit (ActiveMotif, Carlsbad, CA, U.S.A.) according to the manufacturer’s protocol. The obtained supernatant was subjected to a hOGG1 and hAAG enzyme activity assay.

## Conflicts of interest

There are no conflicts to declare.

## Supplementary Material

Supplementary information
