# Supplementary material for: Simultaneous sensitive detection of multiple DNA glycosylases from lung cancer cells at the single-molecule level
Source: Chem Sci. 2017 Nov 7;9(3):712–20. doi: 10.1039/c7sc04296e (PMC5869805; doi:10.1039/c7sc04296e)
Supplement: Supplementary file 1 [file SC-009-C7SC04296E-s001.pdf]

Electronic Supplementary Material (ESI) for Chemical Science.
This journal is © The Royal Society of Chemistry 2017

Electronic Supplementary Information

Simultaneously sensitive detection of multiple DNA glycosylases from lung cancer cells at the single-molecule level

Juan Hu,[a,‡] Ming-hao Liu,[a,‡] Ying Li,[b,‡] Bo Tang,[a,*] and Chun-yang Zhang[a,*]

a. College of Chemistry, Chemical Engineering and Materials Science, Collaborative Innovation Center of Functionalized Probes for Chemical Imaging in Universities of Shandong, Key Laboratory of Molecular and Nano Probes, Ministry of Education, Shandong Provincial Key Laboratory of Clean Production of Fine Chemicals, Shandong Normal University, Jinan 250014, China

b. School of Medicine, Health Science Center, Shenzhen University, Shenzhen 518060, China

Table of Contents

I. Molecular mechanism of DNA glycosylases-mediated cleavage of molecular beacons…………..…..S2

II. Optimization of Experimental Conditions……………………………………………………………S4

I. Molecular mechanism of DNA glycosylases-mediated cleavage of molecular beacons

The hOGG1 is bi-functional DNA glycosylases specific for 8-oxoG base paired with a cytosine (Fig. S1), and exhibits β-elimination AP lyase activity.[1-4] As shown in Fig. S2A, the inactive variant of hOGG1 (Lys 249) retains high-affinity binding to a substrate duplex containing an 8-oxoG/C base pair. The hOGG1 cleaves the N-glycosidic bond using a conserved active site lysine residue (Lys 249) that initiates nucleophilic attack at C1' of the target nucleotide and forms a covalent protein-DNA intermediate (Schiff base intermediate; a covalent Schiff base is formed between the enzyme and C1' of the deoxyribose). The Cy3-labeled molecular beacon releases the damaged base to form an AP site. The Schiff base intermediate can undergo a lyase reaction via β-elimination at the 3'-phosphodiester bond, resulting in the cleavage of DNA phosphate backbone at the AP site. The β-lyase reaction generates a one base gap containing 5'-phosphate (P) and 3'-phopho-α,β unsaturated aldehyde (PA) termini. In addition, endonuclease activity of APE1 occurs at AP site. As a result, the PA termini of β-elimination is processed by APE1, generating 3'-OH termini. Consequently, in the presence of hOGG1, Cy3-labeled molecular beacon is selectively cleaved into two portions due to the combined glycosylase/lyase activity of hOGG1 and endonuclease activity of APE1.

The hAAG is a mono-functional glycosylase specific for deoxyinosine base paired with a thymine (Fig. S1).[5-7] As shown in Fig. S2B, the hAAG specifically binds to DNA containing a target damaged base by inserting a β-hairpin loop into the minor groove of DNA. Glu125 acts as a general base to deprotonate a water molecule, which may serve as a nucleophile to attack the anomeric C1' carbon in an S_N2 catalytic mechanism. In the presence of hAAG, the products are the hypoxanthine and AP site. Subsequently, APE1 performs an incision at the 5' side of the AP site, resulting in a nick in the phosphodiester backbone with 3'-OH and 5'-deoxyribose-5-phosphate (dRP) ends. Consequently, in the presence of hAAG, Cy5-labeled molecular beacon is selectively cleaved into two portions due to the combined glycosylase activity of hAAG and endonuclease activity of APE1.

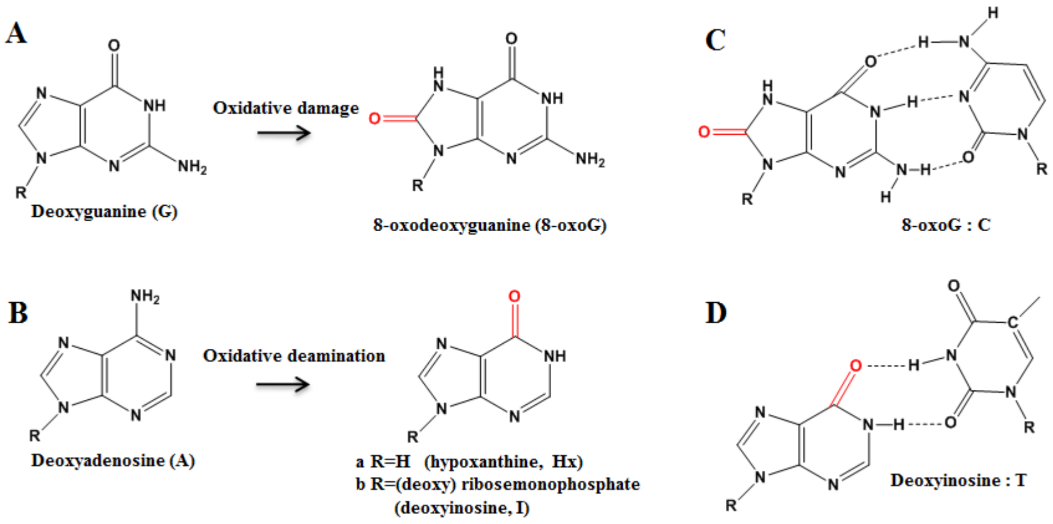

Fig. S1 (A) Chemical structure of 8-oxoguanine (8-oxoG) generated by oxidative damage of DNA by reactive oxygen species. (B) Oxidative deamination of adenosine. (C) Structure of 8-oxoG/C base pair. (D) Structures of deoxyinosine /T base pair.

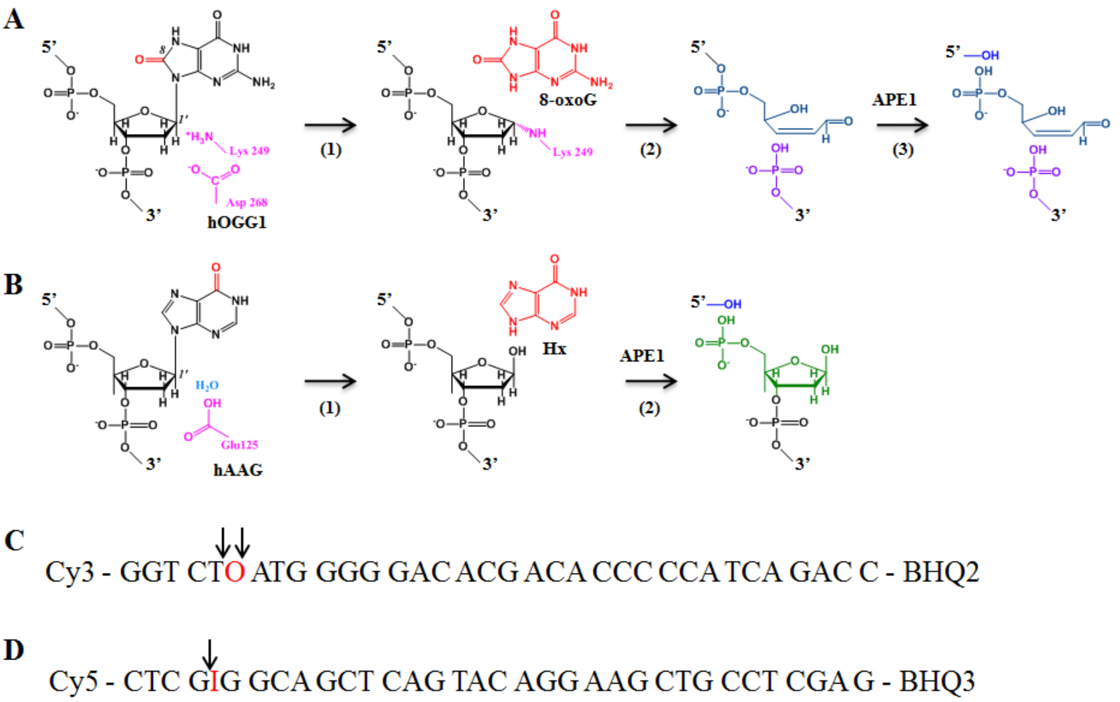

Fig. S2 (A) Mechanism of hOGG1-mediated cleavage of molecular beacon in the presence of APE1. The chemical steps of catalysis include step 1: 8-oxoG base removal and formation of AP site, step 2: β-elimination of 3'-phosphate resulting in formation of the nicked product, step 3: removal of PA termini by APE1. (B) Mechanism

of hAAG-mediated cleavage of molecular beacon in the presence of APE1. The chemical steps of catalysis include step 1: hypoxanthine (Hx) base removal and formation of AP site, step 2: cleavage of the phosphodiester bond on the 5' side at the AP site. (C) Cy3-labeled molecular beacon is 34 nt, which is modified with a 8-oxoG positioned 6 deoxynucleotides downstream of a 5'-terminus. Due to hOGG1-mediated cleavage of molecular beacon in the presence of APE1, one deoxynucleotide is cleaved (Fig. S2A), generating a 5 nt of Cy3-labeled DNA fragment and a 28 nt of BHQ2-labeled DNA fragment. (D) Cy5-labeled molecular beacon is 34 nt, which is modified with a deoxyinosine positioned 5 deoxynucleotides downstream of a 5'-terminus. Due to hAAG-mediated cleavage of molecular beacon in the presence of APE1, one hypoxanthine base is cleaved (Fig. S2B), generating a 4 nt of Cy5-labeled DNA fragment and a ~29 nt of BHQ3-labeled DNA fragment because the cleavage is one base instead of one deoxynucleotide.

II. Optimization of experimental conditions.

To achieve the best performance, we optimized the concentrations of APE1, Cy3-labeled molecular beacon and Cy5-labeled molecular beacon, and the incubation time. Previous research demonstrates that APE1 accounts for >95% of the total AP endonuclease activity in most human cell lines,[8] and has been implicated in the stimulation of catalytic turnover of many human DNA glycosylases (e.g., hOOG1 and hAAG).[9-12] Since APE1 serves to stimulate DNA glycosylases-mediated cleavage of molecular beacons in this assay, its concentration should be optimized. In the presence of hOOG1, Cy3 fluorescence intensity improves with the increasing concentration of APE1 from 0 to 0.1 U/μL, and reaches a plateau at 0.1 U/μL due to either the complete losing of hOOG1 activity or the consumption of all available substrates by hOOG1 (Fig. S3A, green column). Notably, hOGG1-mediated cleavage of Cy3-labeled molecular beacon may occur in the absence of APE1 (Fig. S3A, green column, 0 U/μL), with less Cy3 fluorescence intensity than that generated in the presence of 0.1 U/μL APE1,

suggesting the stimulation of catalytic turnover of hOOG1 by APE1.[9,10] In the presence of hAAG, Cy5 fluorescence intensity enhances with the increasing concentration of APE1 from 0 to 0.1 U/μL, and reaches a plateau at 0.1 U/μL (Fig. S3A, pink column). Notably, hAAG-mediated cleavage of Cy5-labeled molecular beacon cannot occur in the absence of APE1 (Fig. S3A, pink column, 0 U/μL), and 0.1 U/μL APE1 may induce the improvement of Cy5 fluorescence intensity by 7.6-fold because of the stimulation of catalytic turnover of hAAG by APE1.[11,12] Thus, 0.1 U/μL APE1 is used in the subsequent research.

To optimize the concentration of Cy3-labeled molecular beacon for hOOG1 assay, we monitored the variance of $(F - F_0)/F_0$ value with the concentration of Cy3-labeled molecular beacon, where F_0 and F are the the Cy3 fluorescence intensity in the absence and presence of hOOG1 (0.1 U/μL), respectively. As shown in Fig. S3B, the $(F - F_0)/F_0$ value improves with the increasing concentration of Cy3-labeled molecular beacon from 0.2 to 0.3 μM, followed by the decrease beyond the concentration of 0.3 μM. Therefore, 0.3 μM Cy3-labeled molecular beacon is used for hOOG1 assay in the subsequent research.

We further optimized the concentration of Cy5-labeled molecular beacon for hAAG assay by monitoring the variance of the $(F - F_0)/F_0$ value with the concentration of Cy5-labeled molecular beacon, where F_0 and F are the Cy5 fluorescence intensity in the absence and presence of hAAG (0.1 U/μL), respectively. As shown in Fig. S3B, the $(F - F_0)/F_0$ value enhances with the increasing concentration of Cy5-labeled molecular beacon from 0.2 to 0.3 μM, followed by the decrease beyond the concentration of 0.3 μM. Thus, 0.3 μM Cy5-labeled molecular beacon is used for hAAG assay in the subsequent research.

We further employed single-molecule detection to optimize the incubation time of DNA glycosylases-mediated cleavage of molecular beacons. In the presence of hAAG (Fig. S3C), Cy3 counts increase with the incubation time and reach a plateau at 10 min due to either the complete losing of hOOG1 activity or the consumption of all available substrates by hOOG1. In contrast, no significant Cy3 counts are detected in the

absence of hOOG1 even after a long incubation time of 90 min. In the presence of hAAG (Fig. S3D), Cy5 counts increase with the incubation time and reach a plateau at 90 min, while no significant Cy5 counts are detected in the absence of hAAG even after a long incubation time of 180 min. Taking into account the simultaneous detection of hOGG1 and hAAG, we used the reaction time of 90 min in the subsequent research.

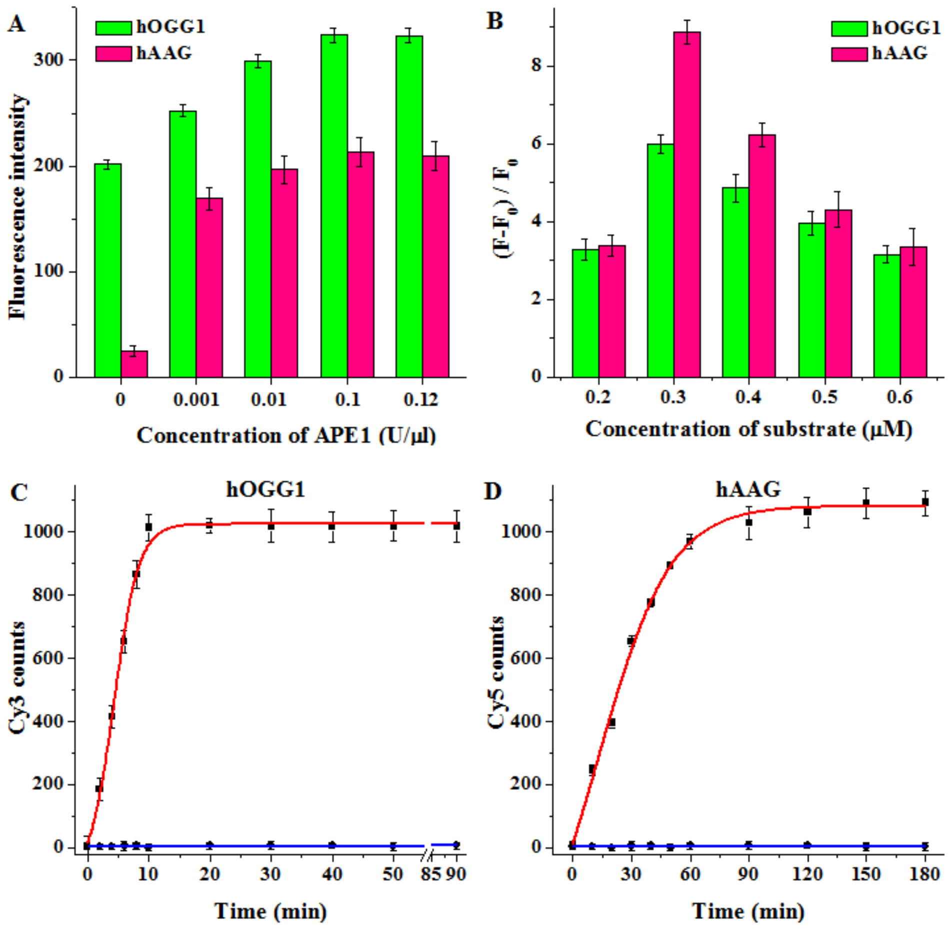

Fig. S3. (A) Variance of fluorescence intensity with the APE1 concentration in the presence of hOGG1 (green color) and hAAG (red color), respectively. The hOGG1concentration is 0.1 U/µL and the hAAG concentration is 0.1 U/µL. (B) Value of $(F - F_0)/F_0$ with the concentrations of Cy3-labeled molecular beacon (green color) and Cy5-labeled molecular beacon (red color), respectively. F and F_0 are the fluorescence intensity in the presence and absence of DNA glycosylases (0.1 U/µL hOGG1 and 0.1 U/µL hAAG), respectively. The concentration of APE1 is 0.1 U/µL. (C) Variance of Cy3 counts as a function of reaction time in the absence (blue line) and presence (red

line) of hOGG1 (0.1 U/μL). The concentration of Cy3-labeled molecular beacon is 0.3 μM, and the concentration of APE1 is 0.1 U/μL. (D) Variance of Cy5 counts as a function of reaction time in the absence (blue line) and presence (red line) of hAAG (0.1 U/μL). The concentration of Cy5-labeled molecular beacon is 0.3 μM, and the concentration of APE1 is 0.1 U/μL. Error bars represent the standard deviations of three experiments.

Notes and references

1 S. D. Bruner, D. P. Norman and G. L. Verdine, *Nature*, 2000, **403**, 859-866.

2 J. C. Fromme, S. D. Bruner, W. Yang, M. Karplus and G. L. Verdine, *Nat. Struct. Biol.*, 2003, **10**, 204-211.

3 T. K. Hazra, A. Das, S. Das, S. Choudhury, Y. W. Kow and R. Roy, *DNA Repair*, 2007, **6**, 470-480.

4 P. C. Anderson and V. Daggett, *J. Am. Chem. Soc.*, 2009, **131**, 9506-9515.

5 P. J. O'Brien and T. Ellenberger, *Biochemistry*, 2003, **42**, 12418-12429.

6 P. J. O'Brien and T. Ellenberger, *J. Biol. Chem.*, 2004, **279**, 9750-9757.

7 D. M. Lyons and P. J. O'Brien, *J. Am. Chem. Soc.*, 2009, **131**, 17742-17743.

8 Y.-J. Kim and D. M. Wilson, *Curr. Mol. Pharmacol.*, 2012, **5**, 3-13.

9 J. W. Hill, T. K. Hazra, T. Izumi and S. Mitra, *Nucleic Acids Res.*, 2001, **29**, 430-438.

10 A. Esadze, G. Rodriguez, S. L. Cravens and J. T. Stiverse, *Biochemistry*, 2017, **56**, 1974-1986.

11 L. Xia, L. Zheng, H.-W. Lee, S. E. Bates, L. Federico, B. Shen and T. R. O'Connor, *J. Mol. Biol.*, 2005, **346**, 1259-1274.

12 M. R. Baldwin and P. J. O'Brien, *Biochemistry*, 2009, **48**, 6022-6033.
